# Effect of acceptance and commitment therapy for adolescent depression: a meta-analysis

**DOI:** 10.3389/fpsyt.2025.1506822

**Published:** 2025-05-19

**Authors:** Xin Yu, Bing Zhao, Tongtong Yin, Haiying Qu, Jingxuan Zhang, Xiaojing Cheng, Xu Chen

**Affiliations:** ^1^ School of Special Education and Rehabilitation, BinZhou Medical University, Yantai, China; ^2^ Department of Psychiatry, Shandong Mental Health Center, Shandong University, Jinan, China; ^3^ Department of Psychiatry, Institute of Mental Health, Occupational Diseases Hospital of Shandong First Medical University, Jinan, China

**Keywords:** acceptance and commitment therapy, adolescent, depression, randomized controlled trial, meta-analysis

## Abstract

**Objectives:**

This study conducted a systematic review of published randomized controlled trials (RCTs) to evaluate the efficacy of acceptance and commitment therapy (ACT) for adolescent depression, while also examining its pertinent characteristics.

**Methods:**

We conducted systematic searches of electronic databases in accordance with PRISMA guidelines to ensure rigorous screening, data extraction, and quality assessment. Additionally, we employed random-effects models and performed subgroup analyses.

**Results:**

A meta-analysis of 25 studies with 2,352 participants showed that ACT significantly reduced depressive symptoms in adolescents. Improvements in psychological flexibility significantly predicted reductions in depression. Subgroup analysis revealed that, at post-test, ACT was significantly more effective than wait-list controls in reducing depressive symptoms, though it was not superior to Treatment As Usual or active treatment groups. Furthermore, offline ACT was significantly more effective than Internet-based ACT in reducing depressive symptoms. Comparisons of intervention settings also indicated that ACT was more effective in group settings than individual ones. However, no significant difference was found between clinical and non-clinical samples.

**Conclusions:**

The current study suggests that ACT effectively reduces depressive symptoms, with psychological flexibility playing a crucial role in this improvement. Intervention forms and sample types should be considered when implementing ACT interventions. Extensive research is still needed for further exploration.

**Systematic review registration:**

https://www.crd.york.ac.uk/PROSPERO/view/CRD42023494677, identifier CRD42023494677.

## 1 Introduction

Depression often originates during adolescence, a period marked by a high incidence and rapidly increasing prevalence. Adolescent depression symptoms differ from those in adults (1), and once established, depression frequently persists throughout an individual’s life (2, 3). The prevalence of suicidal thoughts, intentions, and attempts gradually increases among adolescents struggling with depression (4). Adolescence is a critical period for both physical and mental development. Adolescents face substantial academic pressures and are influenced by various factors, including vulnerabilities, cognitive aspects, interpersonal relationships, and social media use (5–7). Emotions are further influenced by growth and hormonal fluctuations, contributing to emotional instability (8). Furthermore, the COVID-19 pandemic has compounded these challenges (9), as recent research indicates that disruptions to daily routines, increased social isolation, and heightened uncertainty about the future have significantly elevated both the prevalence and severity of depressive symptoms among adolescents (10, 11). Consequently, given the rising incidence of teenage depression and its complex influencing factors, there is an urgent need for research-supported, teen-specific, and cost-effective therapeutic approaches.

Acceptance and Commitment Therapy (ACT) belongs to the third wave of the cognitive-behavioral paradigm and was developed in 1982 by Professor Steven C. Hayes and his colleagues at the University of Nevada, USA (12). Grounded in functional contextualism and relational frame theory, ACT is a psychological approach that emphasizes mindfulness, acceptance, and behavioral change to enhance psychological flexibility (PF) (12, 13). It assists individuals in accepting stressful circumstances, identifying their values, discovering and adapting behavioral techniques, and openly engaging in meaningful activities (13). To cultivate PF, ACT employs six interconnected processes: Acceptance – adopting a positive, non-defensive attitude toward a range of experiences; Cognitive Defusion – adjusting one’s relationship with thoughts, images, and memories so that they can be observed without becoming entangled; Being Present – focusing on the current moment in a non-evaluative manner rather than dwelling on the past or future; Self-as-Context – shifting from an evaluative self-concept to viewing oneself as a context for various psychological events; Values – identifying life directions that impart meaning, regarded as ongoing pursuits rather than finite goals; and Committed Action – translating values into actionable steps across short-, medium-, and long-term objectives (14). In contrast, PF, characterized by cognitive fusion and experience avoidance, stands in direct opposition to this approach (13).

Depressed individuals often become entangled with negative self–evaluations, resulting in narrowed and rigid behavior (15). Prolonged cognitive fusion contributes to experiential avoidance, thereby intensifying and sustaining depressive symptoms (16, 17). ACT provides various exercises aimed at enhancing cognitive defusion, helping individuals alter how they relate to their thoughts, feelings, and bodily sensations. This process promotes PF and plays a key role in reducing depressive symptoms (15). While ACT emphasizes cognitive defusion, Cognitive Behavioral Therapy (CBT) also effectively addresses negative cognitive patterns by challenging and restructuring maladaptive thoughts. However, CBT typically requires higher levels of executive functioning, which can be particularly challenging for adolescents who often experience emotional instability and cognitive overload (18). The efficacy of ACT in treating depression has been well–documented in adults (19). Systematic reviews and meta–analyses have shown that ACT significantly reduces depressive symptoms, resulting in substantial improvements in mental well–being and overall quality of life (20, 21). Similarly, evidence supporting the effectiveness of ACT in adolescents is growing. Recent studies have shown that ACT significantly alleviates depressive symptoms in this population (22). Burckhardt et al. found that ACT not only improves mood but also enhances PF, a crucial factor in helping adolescents navigate developmental challenges and psychosocial stressors (23). These findings suggest that ACT may be particularly beneficial in promoting adaptive coping strategies during periods of emotional turmoil. Despite its positive outcomes, the superiority of ACT over other interventions remains debated. For example, a meta–analysis by López−Pinar, which reviewed randomized controlled trials (RCTs), concluded that ACT did not show a significant advantage over CBT, although it was more effective than treatment as usual (TAU) and inactive controls in reducing depressive symptoms (24). Similarly, Fang reported that ACT was more effective than wait–list or TAU in treating anxiety and depression in children and adolescents, though no significant difference was observed when compared to CBT (25). A review by Knight and Samuel further emphasized that ACT showed positive effects on both anxiety and depression symptoms in school–based intervention programs (26). These findings highlight the versatility of ACT as a therapeutic approach for depression across the lifespan, underscoring its potential to address both clinical and subclinical manifestations of depression in adolescents. While further investigation is needed to clarify its relative efficacy, the growing body of evidence suggests that ACT may be a promising tool for improving mental health outcomes in this population.

ACT, originating from Western culture, emphasizes individual autonomy and choice (15, 27). However, in cultural contexts such as Asian cultures, which emphasize collectivism, the core concepts and methods of ACT may require adaptation to align with the cognitive and affective habits of adolescents (28). Recognizing cultural differences is crucial for tailoring therapies for diverse populations, thereby enhancing efficacy among various social backgrounds. Moreover, the COVID-19 pandemic has exacerbated adolescent mental health issues while simultaneously fostering the expansion of online psychotherapy (29). The shift towards digital solutions underscores the increasing importance of Internet-based therapies. A recent meta-analysis shows that online mental health interventions, including Internet-based Acceptance and Commitment Therapy (IACT), effectively addresses a wide range of mental health conditions in adolescents (30). IACT merges traditional ACT with digital tools like mobile apps, demonstrating promising results in boosting self-acceptance and facilitating behavioral changes. It integrates both face-to-face meetings and app-based activities, enhancing the delivery of ACT strategies. Although only a few meta-analyses have evaluated the effectiveness of IACT in adolescents, the preliminary results are promising. For example, Wang and Fang reviewed online ACT applications and found that while ACT was more effective than inactive controls in improving depressive symptom (31). IACT may effectively in enhancing PF and reducing depressive symptoms in adolescents, as supported by other studies (21, 32). Additionally, many studies confirm that traditional offline ACT effectively reduces depression among adolescents (33, 34). However, some contrasting findings exist, with certain studies indicating reduced treatment effectiveness compared to control treatments in comparative analyses (35, 36). This variety of findings highlights the complexity of measuring therapy outcomes across diverse settings and populations. Consequently, further examination is essential to determine whether ACT or IACT is more effective, especially for adolescents. In conclusion, despite the extensive body of research, a comprehensive assessment of ACT’s impact on adolescent depression levels remains lacking.

Therefore, this study aims to systematically examine the effect of ACT on adolescent depression levels and explore the potential moderating role of PF in depression outcomes, ultimately providing evidence for clinical practice that targets both symptom reduction and the enhancement of PF.

## 2 Methods

The present review and meta-analysis were conducted in accordance with the PRISMA (Preferred Reporting Items for Systematic Reviews and Meta-Analyses) guidelines (37), which are recommended for comprehensive reporting in systematic reviews and meta-analyses. A Protocol for this review was preregistered with PROSPERO (CRD42023494677).

### 2.1 Search strategy

Relevant articles were selected through a comprehensive search of electronic databases, covering the entire period from the inception of each database until August 23, 2024. The search was updated on December 21, 2024, to ensure the inclusion of the most recent studies. The databases used included the Web of Science Core Collection, PubMed, the Cochrane Library, PsycINFO, CNKI, WANGFANG, and Weipu. *The search strategy in the title, abstract, and keywords included the following terms: For intervention, “Acceptance and Commitment Therapy”, “acceptance-based”, or “ACT”; For population, “depression”, “depressive disorder”; For age group, “adolescent”, “teen”, “teenager”, or “adolescence”; For study design, “randomized controlled trial”, “randomised controlled trial”, or “RCT”.*

A comprehensive search was conducted for studies published in peer-reviewed publications in both English and Chinese. Additionally, a manual search was undertaken by examining the reference lists of the identified articles and utilizing the related article attributes in the databases.

### 2.2 Inclusion and exclusion criteria

The PICOS framework emphasizes the elements of Population (P), Intervention (I), Comparison (C), Outcome (O), and Study design (S). For Population (P), adolescents aged 10–18 diagnosed with depression through a DSM-based clinical interview or other standardized diagnostic criteria were classified as clinical samples, whereas those assessed using standardized questionnaires with established reliability and validity were classified as non-clinical samples. For Intervention (I), the intervention groups comprised individuals receiving ACT or Internet-based Acceptance and Commitment Therapy (IACT). For Comparison (C), control groups comprised individuals on a wait-list (WL), those receiving treatment as usual (TAU), those under medical therapy management (MTM), or those undergoing other forms of treatment. For outcome (O), studies were required to report baseline depression scores, with depression levels serving as the primary outcome measure. Studies were required to report mean values, standard deviations, and sample sizes. For Study design (S), this Meta only includes RCTs to ensure the rigor of the intervention effect assessment. The exclusion criteria for this review were as follows: Duplicate publications, studies with designs that do not meet the RCT requirements, and experiments lacking ethical review. Additionally, redundant studies and studies with missing data were excluded to ensure the reliability and accuracy of the review.

### 2.3 Data extraction

The studies were selected based on the PRISMA guidance (38). Two assessors independently reviewed the literature. In case of any disagreement, the assessors engaged in a discussion to resolve the issue. If a consensus was not reached, an additional assessor was consulted. The process of data extraction adhered to the Cochrane principles for systematic evaluation, and the following information was documented: This includes (1) bibliographic details (2); participant count, average age, and standard deviation (3); gender distribution of the sample (female%) (4); details of the interventions in ACT group (number of ACT interventions, duration of a single session, and overall duration) (5); characteristics of the control group (6); outcome measures and duration of follow-up (see **Table 1** for details).

**Table 1:** Data extraction table of study characteristics.

Study	country	Sample size	Age M ± SD	%F	Samples	Format		Setting	Outcome Indicator	Follow-up months
		T/C	T/C			Treat	Control			
Zemestani et al. 2022	Iran	71	15.2 ± 0.40	100	Non-clinical samples Symptoms of anxiety in adolescent females SCID-V	IACT(Online; group)	WT	45-60min/8week	RCADS-C	1month
		24/47	15.2 ± 0.40							
Hayes et al. 2011	Australia	38	14.61 ± 3.10	71%	Clinical samples At least moderate depression DSM-iv	ACT	TAU	8week	RADS-2	3month
		22/16	15.49 ± 1.35			(Offline; group)				
Lappalainen et al. 2023	Finland	232	15.04 ± 0.20	78%	Non-clinical samples Voluntary Participation of School Students Adolescent Psychosocial Interview Template	IACT	WT	45min/9month	DEPS COMPACT	None
		232/116	14.98 ± 0.12			(Online; individual)				
Livheim et al. 2015	Australia	66	15.01 ± 0.12	88%	Non-clinical samples Mild or moderate depression	ACT	TAU	8week	RADS-2 AFQ-Y8	None
		40/26	14.6 ± 1.03			(Offline; group)				
Livheim et al. 2015	Sweden	32	None	72%	Non-clinical samples Adolescents with a score of ≥80%	ACT	TAU	6week	DAS-S AFQ-Y17	None
		15/17				(Offline; group)				
Lappalainen et al. 2021	Finland	243	15.25 ± 0.30	49%	Non-clinical samples Teens with learning difficulties and random selection Adolescent Psychosocial Interview Template	IACT	WT	45min/5week	DEPS AFQ-Y17	None
		161/82	15.27 ± 0.33			(Online; individual)				
Burckhardt et al. 2016	Australia	267	15.29 ± 0.50	39%	Non-clinical samples Voluntary participation in school	ACT+PP	PP	90min/3month	DASS-21	None
		139/128	16.37 ± 0.65			(Offline; group)				
Shen Chunli 2020	China	50	None	54%	Non-clinical samples At least moderate depression	ACT	WT	90min/8week	SDS AAQ-II	None
		25/25				(Offline; group)				
Yuan Bin et al. 2022	China	80	11.69 ± 2.31	46%	Clinical samples Depressive disorder CCMD-III	ACT	WT	None	DSRSC	None
		40/40	12.33 ± 3.67			(Offline; group)				
Situ Yuyi 2022	China	60	19.5 ± 4.10	40%	Clinical samples Depressive disorder Not explicitly stated	ACT+MTM	MTM	60-90min/4week	HAMD	None
		30/30	20 ± 4.00			(Offline; group)				
Tian Manyu et al. 2023	China	121	14.93 ± 1.34	74%	Clinical samples Depressive disorder DSM-IV	ACT+MTM	MTM	90-120min/2week	HAMD AAQ-II	None
		61/60	15.2 ± 1.58			(Offline; group)				
Zhou Qing 2022	China	57	None	93%	Non-clinical samples School students	ACT+TAU	TAU	60-90min/4month	SDS AFQ-Y8	1month
		28/29				(Offline; group)				
Burckhardt et al. 2017	Australia	48	15.64	42%	Non-clinical samples School students	ACT	WT	25min/7week	DASS-21	1Week
		17/31				(Offline; group)				
Karimi et al. 2018	Iran	30	None	100	Non-clinical samples females students Adolescents with a score of ≥70%	ACT	WT	60min	DASS-21	45 dayes
		15/15				(Offline; group)				
Moghanloo et al. 2015	Iran	34	10.35 ± 2.91	66%	Non-clinical samples Diabetic children	ACT	WT	None	RCDS	None
		17/17	10.59 ± 3.16			(Offline; group)				
Guerrini et al. 2022	Italy	34	15.5 ± 1.37	79%	Non-clinical samples obese adolescents	ACT	WT	3 week	DASS-21 AFQ-Y	None
		17/17	15.6 ± 1.06			(Offline; group)				
Alho et al. 2022	Finland	72	13.44 ± 1.13	63%	Non-clinical samples type 1 diabetes adolescents	ACT	TAU	90 min/2 weeks	RBDI	None
		36/36	13.36 ± 1.22			(Offline; group)			DAAS	
Shabanl et al. 2019	Iran	44	15.6 ± 1.43	45%	clinical samples anxiety DSM-V	ACT+SSRI	MTM(CBT+SSRI)	60min	CDI	3 Months
		22/22	14.95 ± 1.78			(Offline; group)			AFQ-Y8	
Petersen et al. 2023	United States	26	15.7 ± 1.60	73%	Non-clinical samples Anxiety	ACT	WT	60 min/8 weeks	CES-D AFQ-Y	1 month
		13/13	15.6 ± 1.10			(Offline; group)				
Petersen et al. 2024	United States	30	14.5 ± 1.60	67%	Non-clinical samples Anxiety	ACT	WT	50min/14weeks	CES-D AFQ-Y8	10 Weeks
		15/15	13.8 ± 1.50			(Online; individual)				
Wicksell et al. 2009	Sweden	32	14.8 ± 2.40	78%	Non-clinical samples adolescents with pain duration of more than 3 months	ACT	MTM(MDT)	60 min/4 months	CES-DC	1month
		16/16				(Offline; group)				
Van der Gucht et al. 2017	Belgium	586	17 ± 0.67	53%	Non-clinical samples	ACT	WT	120 min/4 weeks	YSR AFQ-Y	12 months
		288/298				(Offline; group)				
Babaie et al. 2019	Iran	20	None	50%	Non-clinical samples adolescents with stuttering	ACT	WT	90min	DASS-21	None
		10/10				(Offline; group)				
Swain et al. 2015	Australia	49	13.8 ± 1.40	63%	clinical samples anxiety DSM-IV	ACT	WT	90min	CDI AFQ-Y	3 months
		16/33				(Offline; group)				
Talaeizadeh et al. 2020	Iran	30	None	100	Non-clinical samples Girls	ACT	WT	1 month	Beck	None
		15/15				(Offline; group)				

T=Treatment groups; C=control groups; TAU: Treatment as Usual; WT: waiting therapy; MTM: medical therapy management; %M=Proportion of male; DSM-IV: The Diagnostic and Statistical Manual of Mental Disorders published by the American Psychiatric Association, fourth edition; DSM-V: The Manual for Diagnosis and Stats of Minal Disorder, ed. V; CCMD-3: The Chinese Standard for Anxiety and Depression for Children (CCMD-3); SCID-V: Diagnostics and Interviews in the Manual developed according to the DSM V; DSM-iv: Structured Clinical Interview with DSM-4; DASS: Depressive Anxieties and Stress Statistics; SDS: Self-Evaluation of Depression; RCADS-C: The Depression Scoreboard for Children’s and Young People’s Anxious and Depressives; RADS-2: The Reno Teenage Depression Table; DEPS: Depression table prepared by Salokangas et al; DSRSC: Self-assessment table for childhood depression disorders; HAMD: Hamilton Depression Ta.; RBDI: Revised Beck Depression Inventory; CDI: Children’s Depression Inventory; YSR: The Youth Self Report; DAAS: The Diabetes Acceptance and Action Scale for Children and Adolescents; AFQ-Y: Avoidance and Fusion Questionnaire for Youth;MDT: Multidisciplinary treatment and amitriptyline

### 2.4 Risk of bias assessment

Two independent researchers assessed the methodological quality of the included RCTs using the Cochrane Collaboration Risk of Bias Tool 2.0 (ROB 2) (39), which evaluates aspects such as random sequence generation, allocation concealment, blinding of participants and investigators, blinding of outcome assessment, completeness of outcome data, and selective reporting. The risk of bias in each domain was categorized as low, high, or unclear, based on the criteria in the Cochrane Collaboration Handbook. The screening process was conducted in two phases: initial screening of title and abstracts and subsequent screening of full texts. Two researchers (XY and BZ) independently performed both screenings, recording the reasons for exclusion at each stage. In the initial screening phase, the researchers assessed 295 articles, achieving good consistency with Cohen’s kappa = 1. In the subsequent phase, the remaining 34 full-text articles were independently reviewed, with similarly high consistency (Cohen’s kappa = 1). In addition, the risk of publication bias was assessed by visual inspection of the funnel plots, the Trim and Fill test, and the Egger test.

### 2.5 Statistical analysis

The statistical analysis was conducted through a meta-analysis of the included studies using RevMan 5.4 and Stata 15.0 software. In this study, we clearly defined the primary outcome as adolescent depression levels both post-intervention and at follow-up, with PF as the secondary outcome measure.

For each study, variance and standard error were calculated based on its sample size, effect size, and reported standard error. To ensure rigorous statistical analysis, we used the standardized mean difference (SMD) to represent effect sizes when the same outcome was assessed using different scales. According to Cohen’s rule of thumb for interpretation, an effect size of 0.2 or smaller is considered small, between 0.2 and 0.5 is considered medium, and an effect size of 0.8 or larger is considered large (40, 41).

Heterogeneity was assessed using both the I² statistic and the Q statistic. The I² statistic reflects the proportion of variability in the overall effect size that is attributable to heterogeneity, ranging from 0% to 100%. Specifically, heterogeneity is categorized as low (0% < I² < 25%), moderate (25% ≤ I² < 50%), high (50% ≤ I² < 75%), and significant (75% ≤ I² < 100%). When heterogeneity is low (I² < 25%), a fixed-effects model is applied; when heterogeneity is high (I² ≥ 50%) and multiple potential factors are present, a random-effects model is used. The *Q* test further supports the model choice: if the Q test is not significant (*p > 0.05*), a fixed-effects model is appropriate for low heterogeneity; if the *Q* test is significant (*p ≤ 0.05*), a random-effects model is warranted. Subgroup analyses were conducted when significant heterogeneity was present. The combined effect size (ES) was calculated and evaluated statistically, and the outcome measures in this study are expressed as weighted mean differences (MD) along with their 95% confidence intervals (95% CI).

To assess whether PF can predict the impact on depression levels, we conducted a meta-regression analysis, using PF as the predictor and depression levels as the dependent variable. Meta-regression was also employed to evaluate the potential impact of continuous variables, such as average age and the percentage of females in the sample. Additionally, subgroup analyses were performed based on the following variables: intervention type (e.g., offline ACT vs. IACT), control group type (WL vs. TAU vs. MTM), sample type (clinical vs. non-clinical), and intervention setting (group vs. individual).

## 3 Results

### 3.1 Selection of studies

**Figure 1:**
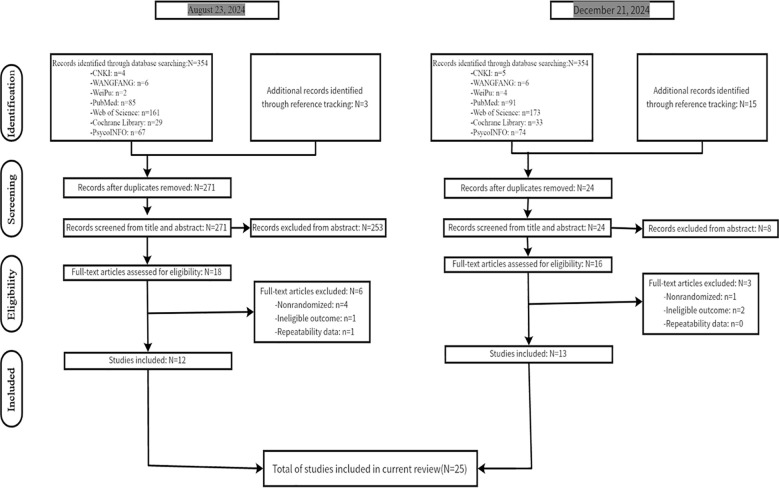
PRISMA flow diagram of the study selection process.

**Figure 1** presents a flow diagram illustrating the article selection process—from the initial search to final inclusion—in accordance with PRISMA guidelines. The initial database search yielded 354 articles, with an additional 3 articles identified through reference tracking. After removing 86 duplicate records, 271 articles remained. Following a screening of titles and abstracts, 18 studies were selected for full-text review. Furthermore, an additional search conducted on December 21, 2024, yielded 13 more studies following full-text review. Notably, one trial—a PhD dissertation (42) —included an ACT intervention section that was consistent with its earlier published, in-print article (43). Consequently, the final selection comprised 24 papers that met the inclusion and exclusion criteria.

Of the 24 publications considered, one article by Livheim et al. (43) reported two separate randomized controlled trials conducted in different countries. Consequently, this article was treated as two distinct studies, bringing the total number of studies to 25. (see **Figure 1**)

### 3.2 Characteristics of the included studies

Twenty-five studies, published between 2009 and 2024, were included in the meta-analysis; all of them were RCTs. These studies comprised 5 Chinese-language papers (44–48) and 20 English-language papers (23, 43, 49–65), involving a total of 2352 participants. Regarding sample type, 6 studies were conducted on clinical samples (45, 46, 48, 51, 58, 64), while 19 studies targeted non-clinical samples (23, 43, 44, 48–50, 52–57, 59–65). Among these, 4 studies focused on IACT (50, 52, 53, 59), while 21 studies focused on offline ACT (23, 43–49, 51, 54–58, 60–65). The control groups varied across studies: Some studies used TAU (23, 43, 47, 51, 56, 57), while other control conditions included WT (44, 48–50, 52–55, 59, 61–65) and MTM (45, 46, 58, 60). In terms of intervention setting, 3 studies delivered interventions on an individual basis (52, 53, 59), whereas the remaining studies were conducted in group settings. The duration of a single session varied between 25 and 120 minutes; notably, 6 studies did not provide information on the duration of a single session (43, 48, 49, 51, 62–64), and 6 studies did not disclose the overall intervention duration (48, 55, 58, 62). Regarding follow-up assessments, 10 studies included a follow-up period (47, 50, 51, 54, 55, 58–61, 65), while 2 studies mentioned a follow-up period but did not provide detailed and comprehensive follow-up data (43, 64) (see **Table 1**).

### 3.3 Risk of bias assessment of included studies

**Figure 2:**
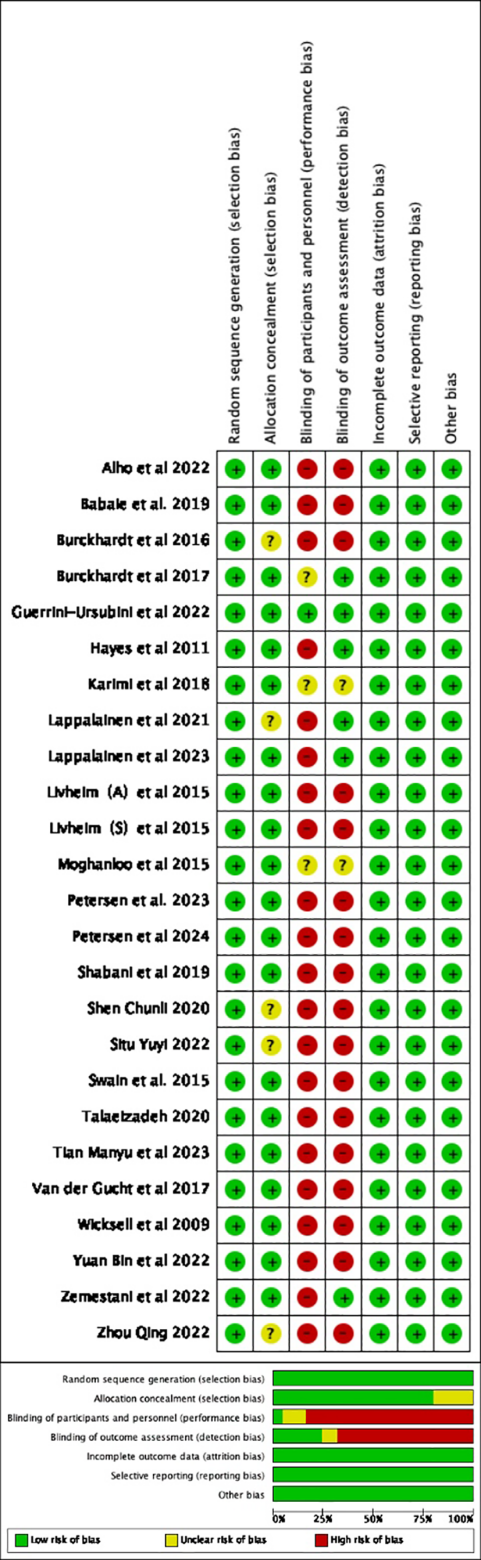
Risk of bias summary.

Summary assessments of the risk of bias, including results from 25 studies, are presented in **Figure 2**. All of these studies were RCTs, with no baseline differences between the experimental and control groups. The studies reported the methods and procedures for correctly assigning participants to groups. Six of the 25 studies employed blinded outcome measures (50–54, 56). A double-blind approach was not feasible due to the nature of the ACT intervention, leading to an overall high risk of bias.

**Figure 3:**
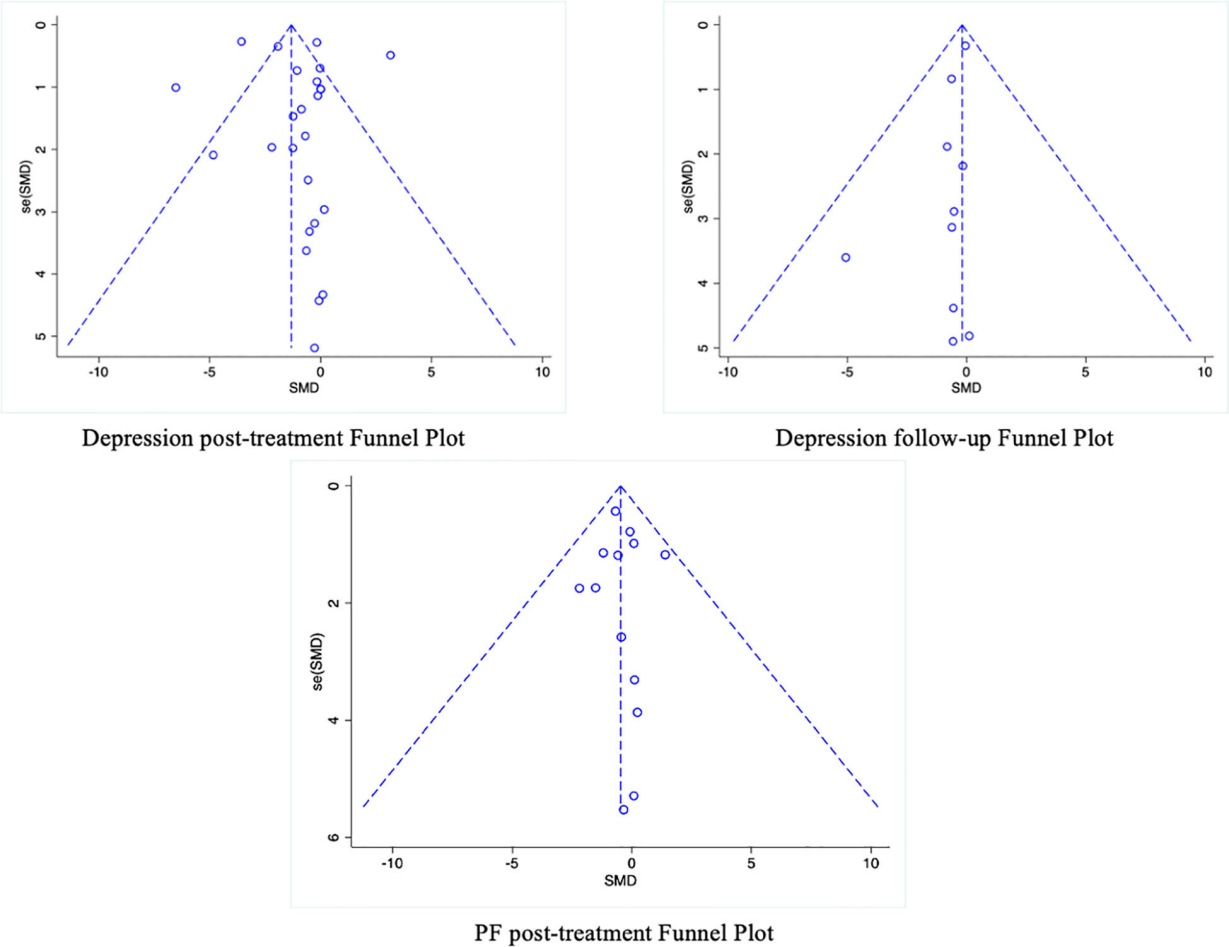
Publication bias of included studies.

Additionally, a funnel plot was used to assess the potential publication bias of each outcome measure. The funnel plots for follow-up depression levels and the secondary outcome of PF showed symmetric scatter distributions, suggesting no publication bias in the analysis. Further Egger tests for each outcome revealed no publication bias (*p* > 0.05). However, the funnel plot for the immediate post-intervention depression levels showed an asymmetric scatter distribution. Further Egger regression tests indicated statistical significance (*p* < 0.05). The Trim-and-Fill method was used to impute missing studies for the post-intervention depression data, and the results were found to be relatively stable, indicating that publication bias had little impact on the study’s findings. The results and the funnel plot are presented in **Table 2**, **Figure 3**.

**Table 2:** Publication bias analyses results.

Outcome	k	Trim and Fill			Egger test	RoB
		Studies imputed	Adjusted SMD	95%CI		
Depression post-treatment	25	0	-1.68	-2.72 to -0.64	-2.54^*^	high
Depression follow-up	10	0	-0.18	-0.75 to 0.39	0.14	high
PF post-treatment	13	0	-0.60	-1.17 to -0.04	-1.57	high

SMD, standardized mean differences; RoB, Risk of bias; ^*^ *p*<0.05, ^**^ *p*<0.01, ^***^ *p<*0.001.

### 3.4 Meta-analysis results

#### 3.4.1 The impact of ACT on depression in adolescents

**Figure 4:**
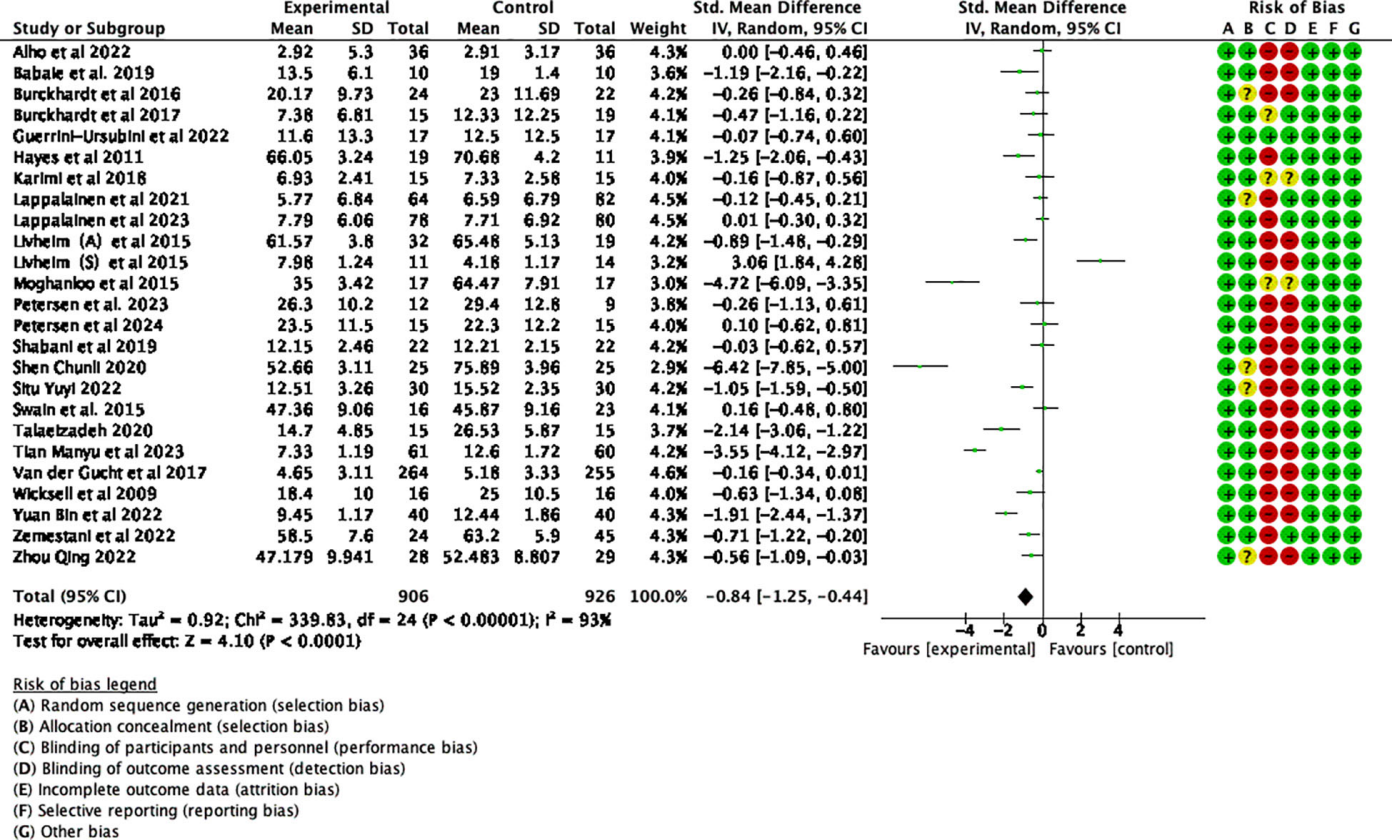
Effect of ACT post-test on depressive symptoms in adolescents after sensitivity analysis.

**Figure 5:**
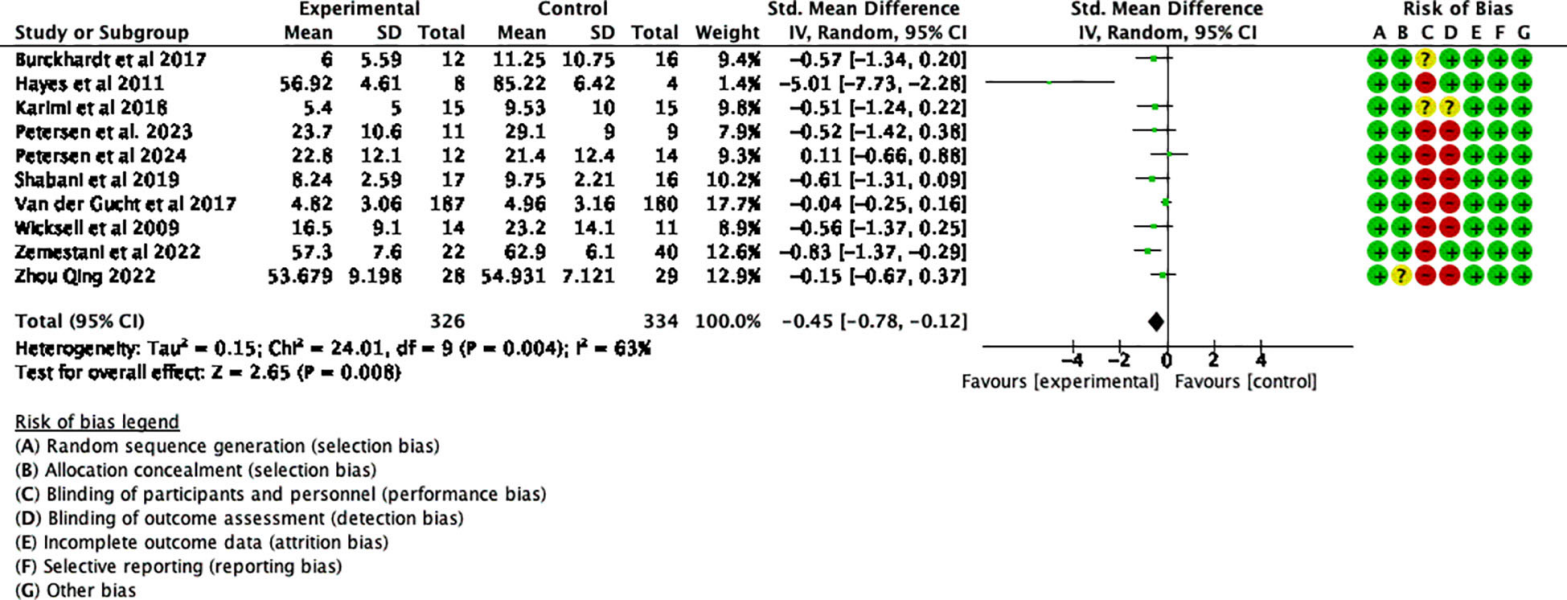
Effect of ACT follow-up test on depressive symptoms in adolescents after sensitivity analysis.

Twenty-five studies examining the impact of ACT included a total of 2352 participants at baseline, with 1832 participants assessed at post-treatment, and 690 participants completing follow-up assessments. Significant statistical heterogeneity was observed among the studies (*p* < 0.00001; I² = 93%). The results are displayed in the forest plots in **Figures 4**, **5**. ACT significantly reduced depression levels upon completion of the intervention compared to the control group (SMD = -0.84, 95% CI: -1.25 ~ -0.44, *p* < 0.0001). Furthermore, ACT showed good maintenance of effect during the follow-up period (SMD = -0.45, 95% CI: -0.78 ~ -0.12, *p* = 0.008).

#### 3.4.2 Meta-regression results

**Figure 6:**
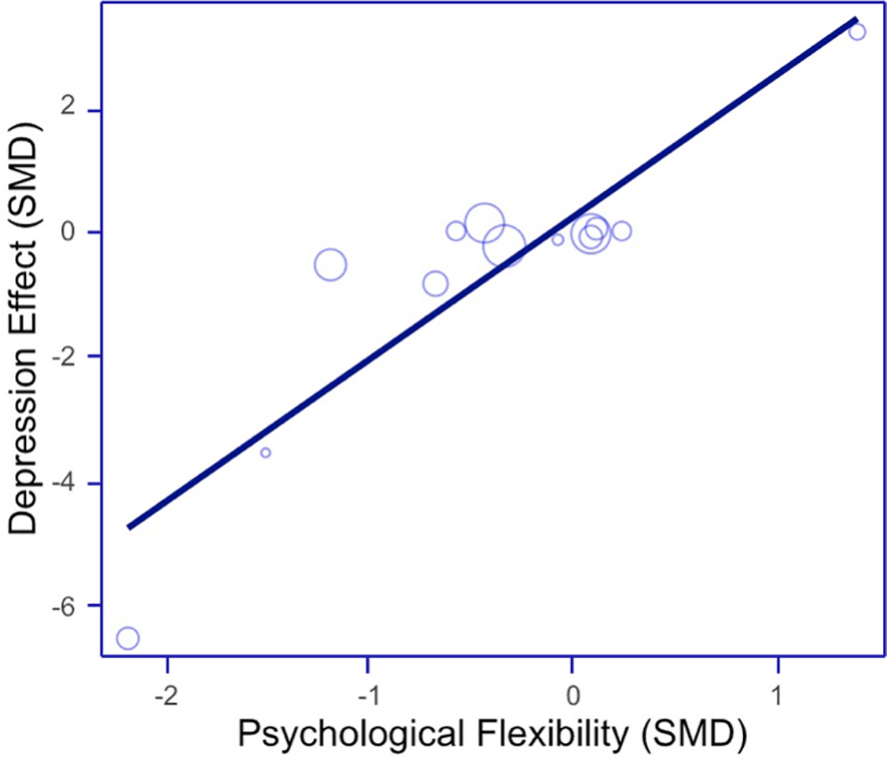
Scatter plot for meta-regression of depression outcome (psychological flexibility as predictor variable).

The main results of the meta-regression are presented in **Table 3**. The results indicate that an increase in PF significantly and positively predicted a reduction in depression, suggesting that higher PF leads to better outcomes on our dependent variables.(See **Figure 6**) The percentage of variance explained was 81% at post-treatment. In contrast, age and the percentage of females did not significantly predict depression levels.

**Table 3:** Results of the meta-regressions.

Dependent variable	predictor	Coefficient	Standard error	*p*-value	R^2^
Depression	Psychological flexibility	2.34	0.17	<0.0001	0.81
	Sample mean age	0.16	0.27	0.57	0.47
	% of females in the sample	0.01	0.02	0.67	0.01

#### 3.4.3 Analysis of heterogeneity

**Figure 7:**
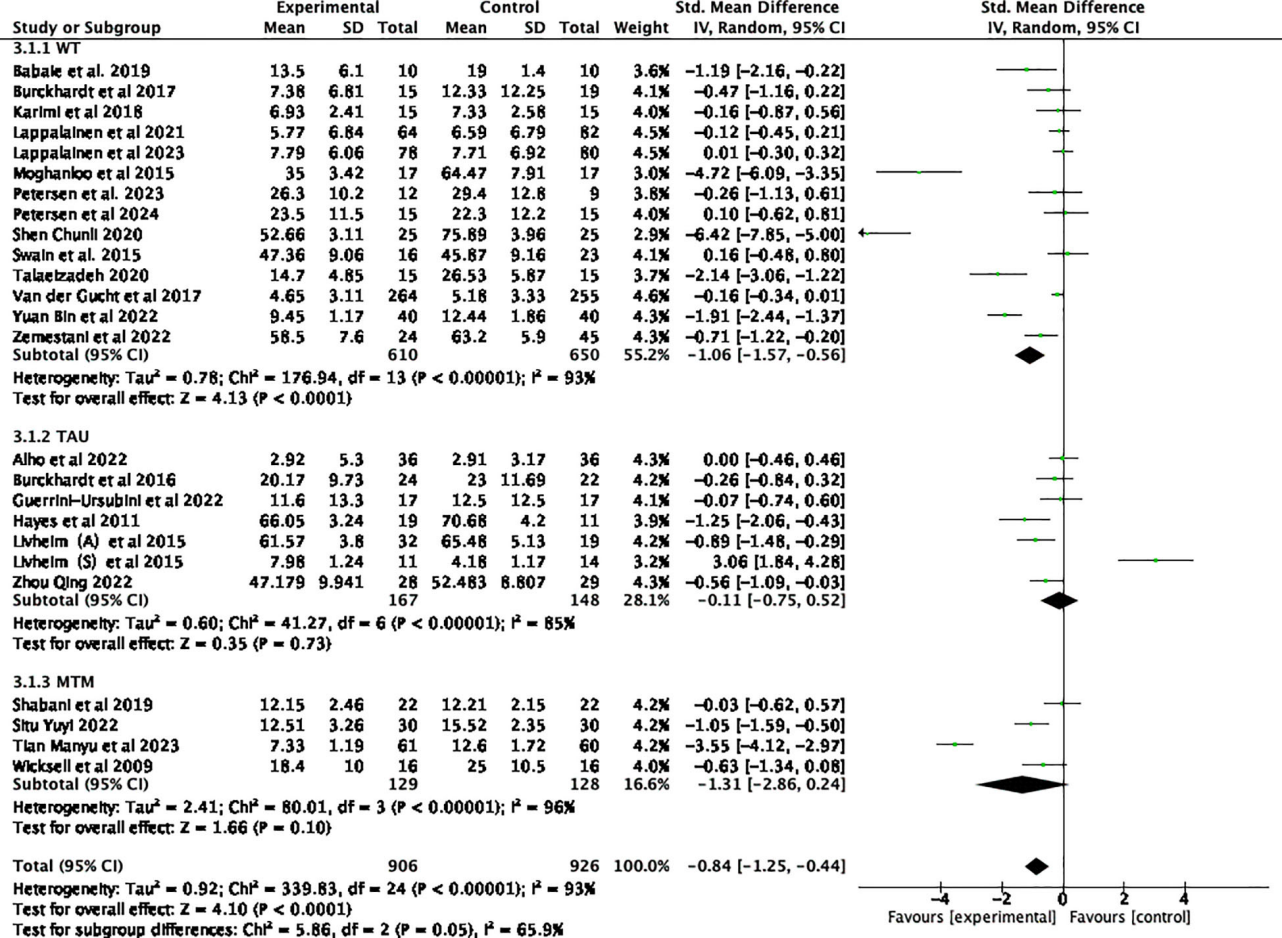
Subgroup analyses of different control treatments.

Subgroup analyses involving various control groups are presented in the forest plots in **Figure 7**. The subgroup analysis revealed that differences among the control groups were statistically significant (*p* = 0.05) and served as a source of heterogeneity (I² = 65.9%). When comparing ACT with WT control groups, an analysis of 14 studies demonstrated that ACT had a statistically significant effect on depressive symptoms (44, 48–50, 52–55, 59, 61–65) (SMD = -1.06, 95% CI: -1.57 ~ -0.56, *p <* 0.0001). However, when compared to TAU (23, 43, 47, 51, 56, 57), ACT did not show a statistically significant effect on adolescent depression (SMD = -0.11, 95% CI: -0.75 ~ 0.52, *p* = 0.73). Similarly, when compared to the medication group (45, 46, 58, 60), ACT was not found to be effective in treating depression (SMD= -1.31, 95% CI: -2.86 ~ 0.24, *p* = 0.10).

**Figure 8:**
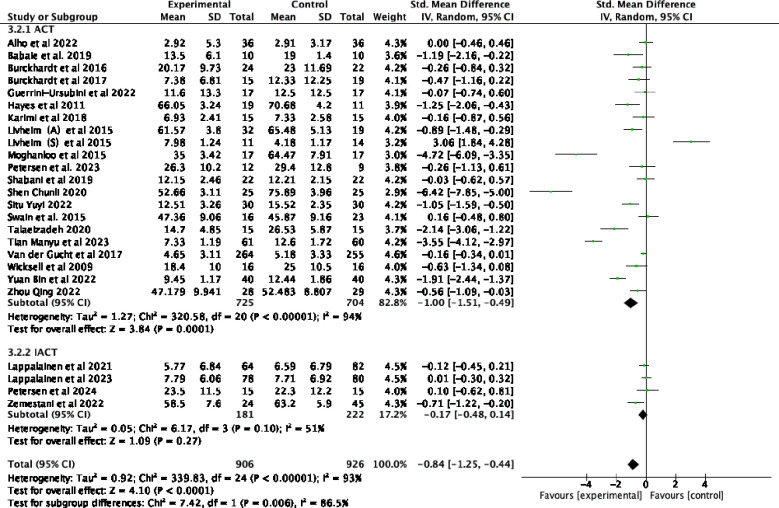
Subgroup analyses of different forms of intervention.

Subgroup analyses, which examined various forms of intervention, revealed that differences among subgroups based on intervention form were statistically significant (*p =* 0.006) and served as a source of heterogeneity (I² = 86.5%). Offline ACT interventions (23, 43–49, 51, 54–58, 60–65) were found to be highly effective (SMD= -1.00, 95% CI: -1.51 ~ -0.49, *p=*0.0001). In contrast, the combined results from 4 online trials (50, 52, 53, 59)demonstrated that IACT treatments were not effective for adolescent depression (SMD= -0.17, 95% CI: -0.48 ~ 0.14*, p* = 0.27). As demonstrated by the forest plots in **Figure 8**, the offline form of ACT was more effective in reducing depression among adolescents.

**Figure 9:**
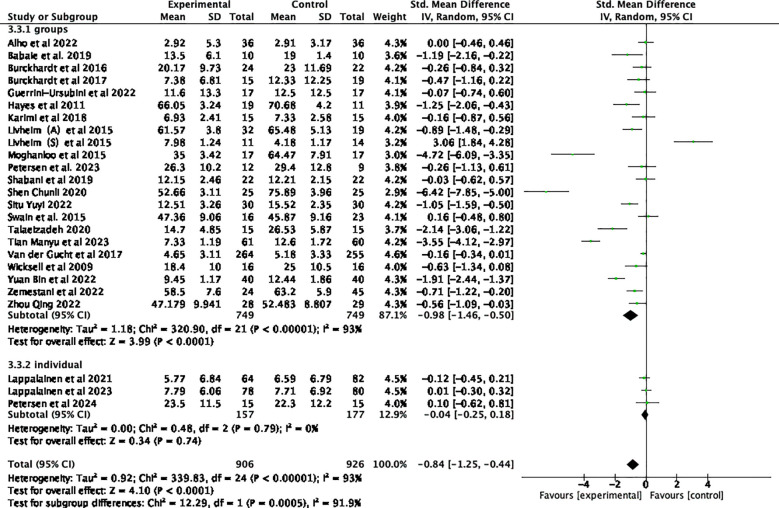
Subgroup analyses of different staffing settings.

Subgroup analyses in **Figure 9** of various forms of staffing settings revealed significant differences across studies (*p* = 0.0005) and served as a source of heterogeneity (I² = 91.9%). The group ACT intervention was found to be highly effective (SMD = -0.98, 95% CI: -1.46 ~ -0.50*, p* < 0.0001) compared to individual interventions (SMD = -0.04, 95% CI: -0.25 ~ 0.18*, p* = 0.74).

**Figure 10:**
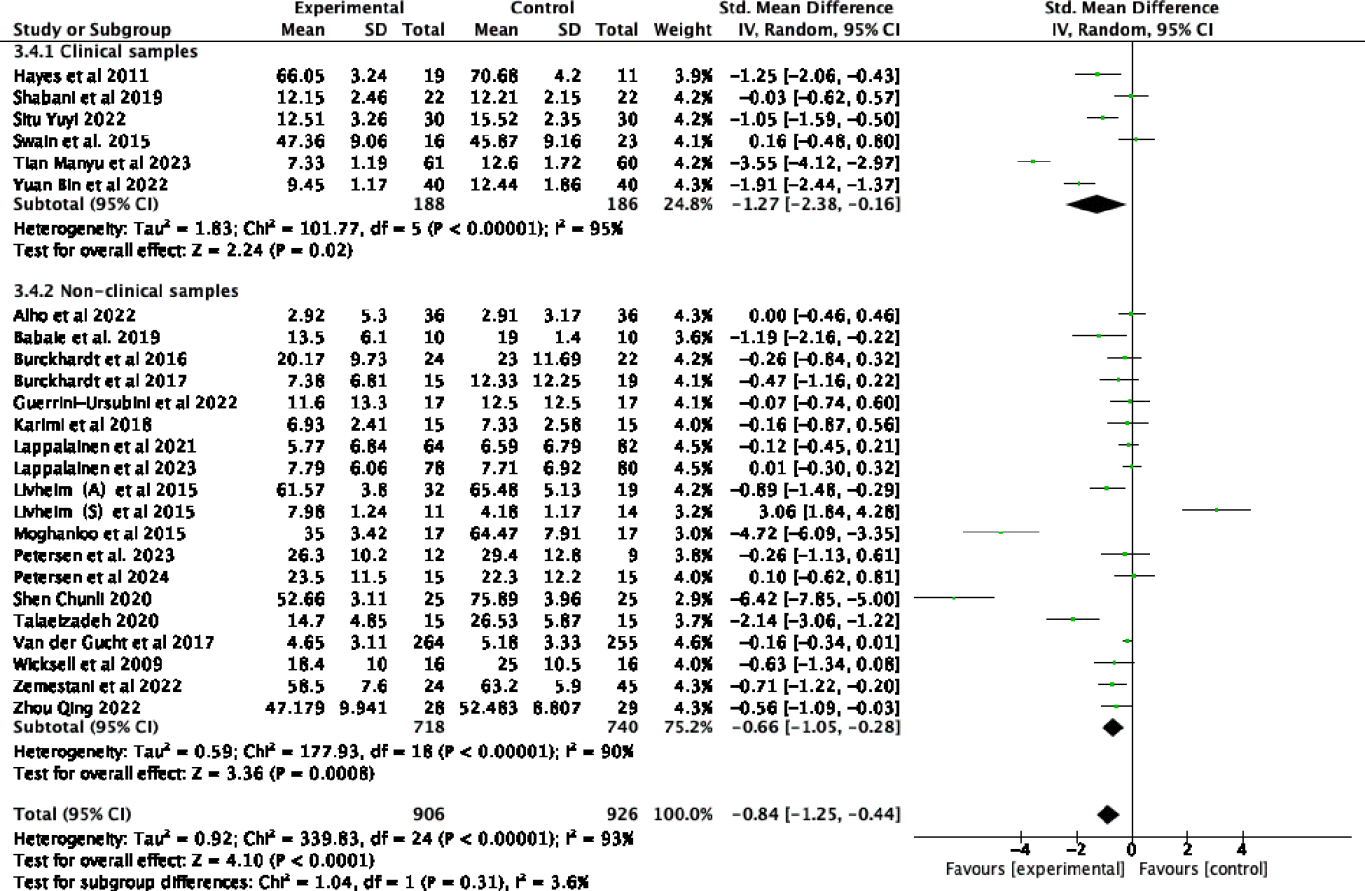
Subgroup analyses of different types of samples.

Subgroup analyses based on various sample types are presented in the forest plots in **Figure 10**. The findings indicated that differences between subgroups based on sample type were not statistically significant (*p* = 0.31) and did not serve as a source of heterogeneity (I² = 3.6%). ACT demonstrated a significant therapeutic effect on a clinical sample of patients with diagnosed depression (45, 46, 48, 51, 58, 64) (SMD = -1.27 95% CI: -2.38 ~ -0.16, *p* = 0.02). Similarly, in the non-clinically diagnosed group, ACT significantly improved depressive symptoms (SMD = -0.66, 95% CI: -1.05 ~ -0.28*, p =* 0.0008).

#### 3.4.4 Effects of ACT on adolescent psychological flexibility

**Figure 11:**
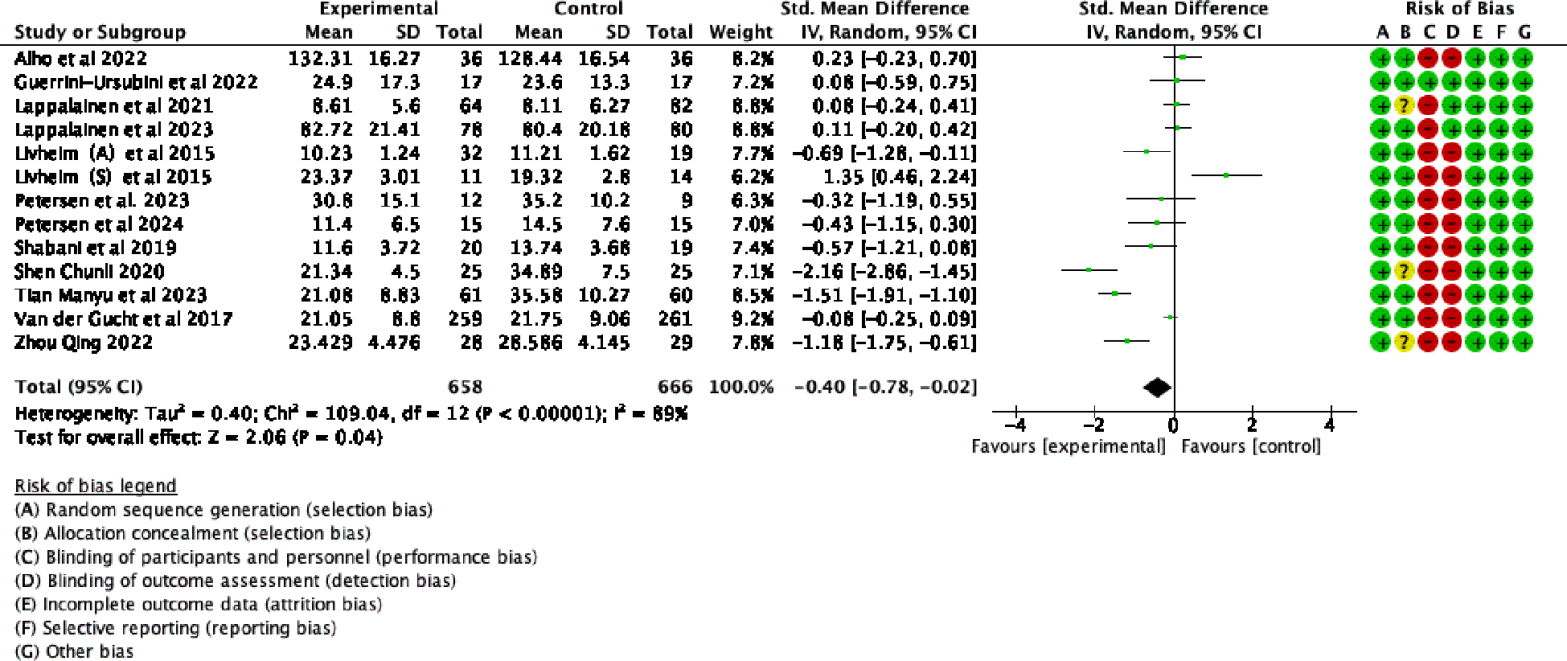
Sensitivity analysis of ACT on psychological flexibility in adolescents.

Thirteen randomized controlled trials demonstrated changes in adolescent PF following ACT, as illustrated in **Figure 11**. The statistical analysis revealed a substantial level of heterogeneity among the studies (*p* < 0.0001; I^2^ = 89%). The meta-analysis, using a random-effects model, revealed statistically significant effect of ACT on adolescent PF (SMD= -0.40, 95% CI: *-0.78 ~ -0.02, p* = 0.04)

## 4 Discussion

### 4.1 Summary of findings

This meta-analysis includes 25 randomized controlled trials that investigated the impact of ACT on depression in adolescents. A total of 2,352 subjects participated in these studies. The results show that ACT is effective in reducing depression levels in adolescents, and PF plays a moderating role in this effect. Through practices such as acceptance, cognitive defusion, and other related techniques, individuals can align their behaviors with their personal values, thereby effectively reducing depressive symptoms (66, 67). *A meta analysis by López-Pinar et al. found that improvements in PF significantly predicted reductions in depressive symptoms and accounted for a substantial proportion of the variance* (24). This finding provides strong empirical support for PF as a core mechanism in ACT interventions. Previous research has shown that the therapeutic effects of ACT are facilitated by enhanced PF (68). Recent studies have also emphasized that ACT not only improves depressive symptoms but also enhances PF (26, 69). Several studies have demonstrated that PF predicts improvements in depressive symptoms.

ACT has been evaluated in 25 randomized controlled trials assessing its efficacy against various control conditions, including wait-list groups, conventional treatments, and medication. These trials included a variety of study designs and participant profiles, offering a comprehensive understanding of ACT’s performance across diverse therapeutic contexts. The analysis of these studies demonstrated that ACT significantly reduces depression levels in adolescents compared to wait-list controls, supporting the findings reported by López-Pinar (24). His study also highlighted ACT’s unique advantages in emotional regulation and behavioral change, suggesting that ACT offers clear benefits over no treatment. ACT works by increasing PF, enabling adolescents to better accept negative emotions and engage in valued actions despite emotional discomfort (70). This is especially beneficial for adolescents who experience intense negative emotions and cognitive distortions, as it helps them separate their sense of self from their emotional states. Similarly, Fang highlighted the efficacy of ACT in children, and our study further supports its effectiveness in treating adolescent depression, suggesting that ACT is broadly applicable across age groups (25). However, when compared to conventional treatments or medication, ACT did not demonstrate statistically significant superiority, indicating that its effects are comparable to those of other active treatments. This conclusion is further corroborated by subsequent studies examining IACT (21, 71), which similarly found no significant differences in outcomes compared to other established therapeutic interventions. Given the variable effectiveness of ACT across different control conditions, a one-size-fits-all approach is unlikely to be optimal for treating adolescent depression. Developing individualized treatment plans that reflect each adolescent’s clinical profile, circumstances, and needs is therefore essential. Tailored care can better address the nuanced challenges adolescents encounter in real-world settings, ultimately improving outcomes and ensuring treatment that is both effective and contextually appropriate.

IACT can mitigate certain drawbacks of traditional manualized interventions, notably their high cost and time-intensive nature. However, this study found that IACT did not outperform offline ACT in reducing depression levels. These findings are consistent with the outcomes of prior meta-analyses (72, 73). First, the therapeutic relationship in online interventions tends to be less robust than in offline, face-to-face interventions (74). Participant persistence is a key factor affecting the effectiveness of both ACT and IACT interventions. In offline ACT interventions, participants typically show higher engagement, with persistence rates exceeding 70%. In contrast, lower persistence in IACT interventions results in less effective outcomes. Lappalainen et al. (53) reported that IACT interventions were effective in reducing depressive symptoms among adolescents who completed at least half of the program. This success in alleviating depressive symptoms is observed in adolescents who adhere to the IACT intervention schedule. However, for the entire randomized sample, there were no significant differences in changes in depressive symptoms between the IACT intervention group and the control group. Therefore, it is essential to improve persistence and motivation among adolescents in IACT interventions to effectively alleviate adolescent depression. Finally, peer support and interaction significantly influence the outcomes of interventions. Peer support is vital for improving the well-being of adolescents with depression (5). Adolescence is characterized by a significant behavioral shift toward increased social interaction, a trend less prevalent in adults (75). Consequently, adolescents tend to seek more peer support and social engagement, and are greatly influenced by peer behaviors (76). Offline ACT primarily consists of group sessions, facilitating substantial peer support and interaction. Therapy participation with peers fosters mutual recognition, shared experiences, and a sense of belonging, thereby enhancing experiential empathy among participants (77, 78). Simultaneously, group therapy participation can reduce feelings of loneliness and provide opportunities to learn new coping strategies and behaviors from peers (4, 7, 37, 79, 80). The studies by Lappalainen et al. on individual interventions (52, 53)indicated no significant improvement in depressive symptoms in their whole randomized samples. Notably, the 2023 study was conducted during the COVID-19 pandemic (52). Adolescents have a strong need for peer contact and social support (78), and the constraints imposed by COVID-19, requiring them to confront significant external challenges, can exacerbate depressive symptoms (37). During the lockdown, with schools closed and a shift to online learning, adolescents experienced diminished social relationships and isolation from their peers (79). Consequently, the application of individual IACT interventions during this period proved ineffective in treating depressive symptoms.

Although no significant difference in ACT’s intervention effects was found between clinical and non-clinical samples, ACT demonstrated a significant effect on adolescent inpatients with depressive disorders, effectively reducing depressive symptoms. Additionally, in non-clinical samples of healthy individuals, ACT has been shown to reduce depression levels. This finding is consistent with previous research, which suggests that individuals with more severe depression show greater potential for improvement following intervention, leading to stronger treatment effects (80). This suggests that ACT may be an effective intervention not only for clinical populations but also for individuals at risk of developing depressive symptoms. The lack of a significant difference between clinical and non-clinical samples may be due to common underlying factors contributing to depression, such as emotional regulation difficulties and low PF, which ACT directly targets (24). However, it is important to consider that the severity of depression in clinical samples may require more intensive or tailored interventions, which could explain the variation in effects between clinical and non-clinical groups. Moreover, the finding that ACT reduces depression in healthy, non-clinical populations supports the growing body of evidence suggesting that even subclinical levels of depressive symptoms can benefit from ACT (25, 26). This highlights the importance of early intervention in preventing the onset of full-blown depressive disorders.

### 4.2 Strengths and limitations

This meta-analysis evaluated the use of ACT in treating adolescent depression and its impact on PF, considering the psychological differences between adolescents and adults. The study also examined the varying effects of interventions across control groups, differences between offline ACT and IACT, and intervention variations among different populations. This study is the latest meta-analysis to analyze the efficacy of ACT in treating adolescent depression. It includes comprehensive subgroup analyses and wide-ranging coverage, thereby enhancing its informativeness.

This study has several significant limitations that impact the interpretation of its findings. Primarily, the meta-analysis included only four IACT studies that met the specified inclusion and exclusion criteria. The small sample size significantly limits the generalizability of the findings. To mitigate this limitation, it is essential to increase the number of studies included in future meta-analyses, thereby enhancing both the generalizability and reliability of the findings. Additionally, the capacity to perform comprehensive subgroup analyses in this study was restricted by the limited number of studies and inconsistent reporting of key variables (participant numbers, intervention durations, symptom severity, and measurement tools). These inconsistencies significantly hinder the effective evaluation of ACT’s efficacy across various subgroups. To systematically and accurately evaluate the efficacy of ACT in treating adolescent depression, researchers must ensure consistent and comprehensive reporting of critical variables. Standardizing these variables can substantially enhance the comparability and generalizability of research findings, thereby yielding more definitive conclusions regarding the effectiveness of ACT.

### 4.3 Implications

Adolescents experience rapid physical and mental development, marked by intense, fluctuating emotions and often displaying contradictory and rebellious behavior. Individuals with depression disorders at varying ages of onset exhibit distinct variations in the manifestation of clinical symptoms. Comparing depression in adulthood to depression in adolescents, it is crucial to consider that physiological and environmental changes throughout adolescence can affect symptoms and progression in this age group. Therefore, analyzing the effectiveness of ACT on adolescent depressive symptoms is essential. This meta-analysis demonstrates ACT’s capability in addressing these symptoms, offering valuable insights for psychologists, therapists, and psychiatrists involved in adolescent depression interventions. It emphasizes the importance of incorporating interactive group settings to boost motivation among adolescents, particularly in IACT programs, to enhance their engagement and persistence in the therapy. Moreover, the findings of this study broaden the applicability of ACT, showing its effectiveness not only in alleviating depressive symptoms among the general adolescent population but also in providing significant benefits for those already experiencing depression. By highlighting the advantages of group interactions and the extended impact of ACT, this research contributes to the field by suggesting practical approaches for improving mental health interventions for young people.

For future research aimed at optimizing ACT effectively, comprehensive reporting of treatment details is crucial. This will enable researchers to draw reliable conclusions on how to maximize the impact of ACT. Additionally, more extensive randomized controlled trials are needed to investigate the effectiveness of both traditional ACT and IACT. Such studies are essential for determining the efficacy of these interventions across diverse settings and populations. Intervention strategies must be thoughtfully tailored to account for the unique physiological and psychological characteristics of adolescents, as well as the environments in which they live. This targeted approach not only enhances the relevance of the interventions but also improves their overall effectiveness. Future research should further explore the relationship between the intervention mechanisms of ACT and depressive symptoms. By focusing on these areas, future research and clinical practice can significantly improve the outcomes of ACT interventions for adolescents, ultimately leading to more sustainable and impactful treatment strategies.

## 5 Conclusions

ACT has been demonstrated to be highly effective in reducing depressive symptoms in adolescents compared to wait-list control groups. However, when compared to active treatment groups, the effects of ACT are not statistically significant. Furthermore, group-format interventions conducted offline are more effective in alleviating depressive symptoms in adolescents than IACT. This study also confirms that PF plays a crucial role in the effectiveness of ACT in improving adolescent depression.

## Data Availability

The original contributions presented in the study are included in the article/supplementary material. Further inquiries can be directed to the corresponding author/s.
